# Supplementary material for: Prenatal Exposure to Traffic-Related Air Pollution and the DNA Methylation in Cord Blood Cells: MOCEH Study
Source: Int J Environ Res Public Health. 2022 Mar 10;19(6):3292. doi: 10.3390/ijerph19063292 (PMC8949945; doi:10.3390/ijerph19063292)
Supplement: Supplementary file 1 [file ijerph-19-03292-s001.zip › EWAS MOCEH_supplementary tables_220204.pdf]

Supplementary Materials for “”

Supplementary Table S1. Numbers of samples excluded in each step of the methylation CpG data quality control (QC) procedures. For leukocyte composition outliers, numbers of samples judged as outliers by each of the cell type are listed separately. NK, natural killer cell. nRBC, nucleated red blood cells.

[1] Cord-200, chip analyzed in 2018, was excluded beforehand because the infant sex information was omitted for this sample.

	**All**	**Male**	**Female**
**All samples**	383[1]	194	189
**Bead array metrics**	3	3	0
**Sex mismatch**	6	4	2
**SNP outlier**	3	1	2
**SNP duplicate**	1	-	1
**PC outlier**	0	0	2
**Leukocyte composition outlier**	CD4: 2<br>B cell: 1<br>NK: 1<br>nRBC: 2	CD4: 1<br>CD8: 1<br>B cell: 1<br>NK: 2<br>nRBC: 1	nRBC: 1
**Total bad samples**	19	14	8

Supplementary Table S2. Summary of the genotype data quality control steps. The number of increase or decrease in subjects or variants are shown.

	Subjects	Variants
Initial data	383	766,278
Individual QC		
Genotype missing rate > 5%	-3	
Identity-by-state (IBS) matrix > 0.8	-2	
Outliers from multidimensional scaling (MDS) plots	(None)	
Variant QC		
Genotype missing rate > 5%		-26,193
Hardy-Weinberg equilibrium p-values < 10^{-5}		-3,579
Minor allele frequency (MAF) < 5%		-374,337
Non-autosomal variants		-10,420
Data for phasing	378	352,199
Imputation (with 1000G phase3 reference panel)		+12,305,067
Variant QC (after imputation)		
Genotype missing rate > 5%		-2,070,571
Hardy-Weinberg equilibrium p-values < 10^{-5}		-81
Minor allele frequency (MAF) < 5%		-6,856,710
Final data	378	3,729,904

Supplementary Table S3. Differentially methylated positions (DMPs) detected by limma analyses for NO2 exposure during each pregnancy period.

CpG ID	Log-FC (Whole pregnancy)	p-value (Whole pregnancy)	Log-FC (Early pregnancy)	p-value (Early pregnancy)	Log-FC (Mid-pregnancy)	p-value (Mid-pregnancy)	Log-FC (Later pregnancy)	p-value (Later pregnancy)	Gene annotation	CpG island annotation
All infants										
cg06517429	0.0619	**$4.09*10^{-9}$**	0.0520	$9.91*10^{-8}$	0.0585	**$5.87*10^{-9}$**	0.0480	$6.43*10^{-7}$	*CASP7* 5' UTR / 1st exon	chr10:115439007-115440196 island
cg06772824	0.0313	**$2.18*10^{-8}$**	0.0256	$8.03*10^{-7}$	0.0320	**$1.88*10^{-9}$**	0.0223	$1.30*10^{-5}$	*FAM176A* TSS200 / 5'UTR	chr2:75787717-75788312 island
cg03233931	0.0178	$6.08*10^{-8}$	0.0145	$1.94*10^{-6}$	0.0179	**$1.02*10^{-8}$**	0.0131	$1.27*10^{-5}$	*LRRC20* TSS200 / 5' UTR / body	chr10:72141560-72142637 island
cg18367020	0.0305	$1.88*10^{-7}$	0.0223	$3.94*10^{-5}$	0.0312	**$2.21*10^{-8}$**	0.0243	$5.27*10^{-6}$	*CCT7* TSS200 *C2orf7* TSS1500	chr2:73460085-73461867 island
cg24053061	0.0374	$2.67*10^{-7}$	0.0299	$8.92*10^{-6}$	0.0386	**$2.30*10^{-8}$**	0.0265	$6.37*10^{-5}$	*RIC8B* body	chr12:107167762-107168747 S-shore
cg23560755	0.0262	$1.19*10^{-7}$	0.0214	$2.99*10^{-6}$	0.0263	**$2.37*10^{-8}$**	0.0190	$2.71*10^{-5}$	*SORBS3* body	chr8:22422534-22423702 N-shelf
cg08060718	-0.0219	$2.87*10^{-7}$	-0.0154	$1.05*10^{-4}$	-0.0227	**$2.46*10^{-8}$**	-0.0178	$4.87*10^{-6}$	-	chr2:241640409-241641359 island
cg13422881	0.0181	$2.21*10^{-6}$	0.0111	$1.87*10^{-3}$	0.0202	**$2.48*10^{-8}$**	0.0147	$2.59*10^{-5}$	*ERCC3* TSS200	chr2:128051347-128051857 island
cg23162960	0.0229	$1.26*10^{-7}$	0.0188	$2.91*10^{-6}$	0.0229	**$2.69*10^{-8}$**	0.0169	$1.96*10^{-5}$	*FAF2* TSS200	chr5:175874629-175875428 island
cg24659093	0.0323	$5.81*10^{-7}$	0.0235	$8.99*10^{-5}$	0.0340	**$3.13*10^{-8}$**	0.0243	$3.89*10^{-5}$	PTPMT1 5' UTR / 1st exon	chr11:47586848-47587608 island

cg01106926	0.0301	$1.35*10^{-6}$	0.0223	$1.17*10^{-4}$	0.0325	**$4.14*10^{-8}$**	0.0216	$1.47*10^{-4}$	-	chr4:13537646-13537973 S-shore
cg04837922	0.0176	$2.13*10^{-6}$	0.0123	$3.68*10^{-4}$	0.0192	**$4.85*10^{-8}$**	0.0131	$1.15*10^{-4}$	-	chr5:139125902-139127711 island
cg05736236	0.0366	$4.60*10^{-7}$	0.0323	$1.37*10^{-6}$	0.0375	**$5.35*10^{-8}$**	0.0234	$4.21*10^{-4}$	*EPHA4* body	chr2:222436034-222438941 N-shore
cg01756814	0.0217	$1.76*10^{-7}$	0.0191	$6.96*10^{-7}$	0.0215	**$5.59*10^{-8}$**	0.0151	$7.58*10^{-5}$	*ELOVL6* TSS200	chr4:111119187-111120084 island
cg14547404	0.0336	0.0145	0.0348	$6.07*10^{-3}$	0.0228	0.0825	0.0299	0.0168	*ARHGAP22* TSS1500 / body	chr10:49863620-49864601 N-shore
cg08906307	0.0543	$1.05*10^{-7}$	0.0413	$1.37*10^{-5}$	0.0497	$3.40*10^{-7}$	0.0498	$8.12*10^{-8}$	-	chr1:221064889-221065600 island
cg19190403	-0.0411	$9.45*10^{-8}$	-0.0353	$6.97*10^{-7}$	-0.0388	$1.35*10^{-7}$	-0.0312	$9.08*10^{-6}$	-	-
cg04129282	0.0280	$5.58*10^{-6}$	0.0216	$1.55*10^{-4}$	0.0315	$7.48*10^{-8}$	0.0180	$1.37*10^{-3}$	*WDR93* TSS1500 *PEX11A* body	chr15:90233530-90234083 island
cg16274061	0.0346	$2.13*10^{-6}$	0.0308	$4.85*10^{-6}$	0.0356	$3.04*10^{-7}$	0.0218	$1.07*10^{-3}$	*SAP30L* TSS1500	chr5:153825417-153826526 N-shore
cg00894435	0.0432	$1.49*10^{-6}$	0.0365	$1.05*10^{-5}$	0.0449	$1.48*10^{-7}$	0.0285	$5.10*10^{-4}$	*SV2B* TSS1500	chr15:91642908-91643702 island
Male infants										
cg10840526	0.0656	**$3.33*10^{-8}$**	0.0548	$3.70*10^{-7}$	0.0587	$1.67*10^{-7}$	0.0508	$3.04*10^{-6}$	-	chr7:100815484-100816995 N-shelf
cg13137577	0.0261	$2.78*10^{-7}$	0.0164	$4.54*10^{-4}$	0.0271	**$9.43*10^{-9}$**	0.0214	$3.84*10^{-6}$	*ARHGEF17* TSS1500	chr11:73018452-73020563 N-shore
cg24973886	-0.0308	$4.70*10^{-7}$	-0.0194	$5.41*10^{-4}$	-0.0311	**$5.58*10^{-8}$**	-0.0266	$1.60*10^{-6}$	*ZNF286B* body	chr17:18580659-18581065 island
cg02522311	0.0565	$7.03*10^{-8}$	0.0418	$1.33*10^{-5}$	0.0474	$1.91*10^{-6}$	0.0539	**$1.10*10^{-8}$**	*C6orf48* TSS1500 *SNORD48* TSS1500	chr6:31802598-31802823 N-shore
cg03940064	0.0238	$1.37*10^{-7}$	0.0180	$1.34*10^{-5}$	0.0197	$4.27*10^{-6}$	0.0226	**$2.82*10^{-8}$**	*SPATA18* TSS1500	chr4:52917388-52918280 island

cg19390934	-0.0460	$9.01*10^{-7}$	-0.0324	$5.41*10^{-4}$	-0.0407	$4.02*10^{-6}$	-0.0438	$2.19*10^{-7}$	*MTERFD2* body<br>*SNED1* body	-
cg27535616	0.0923	$6.65*10^{-8}$	0.0763	$9.08*10^{-7}$	0.0775	$1.77*10^{-6}$	0.0801	$2.49*10^{-7}$	Unknown	Unknown
Female infants										
cg14547404	0.0631	$2.98*10^{-3}$	0.0613	$2.19*10^{-3}$	0.0558	$6.62*10^{-3}$	0.0485	0.0112	*ARHGAP22*<br>TSS1500 / body	chr10:49863620-49864601<br>N-shore
cg00670246	0.0743	0.0110	0.0689	0.0125	0.0697	0.0136	0.0544	0.0384	*GRHL1* body	-
cg14262371	0.0176	$6.23*10^{-6}$	0.0143	$1.15*10^{-4}$	0.0199	$9.34*10^{-8}$	0.0114	$1.27*10^{-3}$	*MOV10*<br>5' UTR / 1st exon	chr1:113217475-113218097<br>N-shore

CpG sites that were significant in multi-pollutant models are marked with *italics*. P-values that reached the significance cutoff are highlighted with bold types. TSS1500, ~1.5kb upstream from the transcription start site (TSS). TSS200, ~200kb upstream from the TSS. UTR, untranslated region. N-shelf, ~4kb upstream from the CpG island. N-shore, ~2kb upstream from the CpG island. S-shore, ~2kb downstream from the CpG island.

Supplementary Table S4. Differentially methylated positions (DMPs) detected by limma analyses for PM10 exposure during each pregnancy period.

**CpG ID**	**Log-FC (Whole pregnancy)**	**p-value (Whole pregnancy)**	**Log-FC (Early pregnancy)**	**p-value (Early pregnancy)**	**Log-FC (Mid-pregnancy)**	**p-value (Mid-pregnancy)**	**Log-FC (Later pregnancy)**	**p-value (Later pregnancy)**	**Gene annotation**	**CpG island annotation**
All infants										
cg14547404	0.2109	$\mathbf{4.13*10^{-12}}$	0.0889	$3.28*10^{-6}$	0.0797	$2.26*10^{-4}$	0.0892	$3.24*10^{-6}$	*ARHGAP22* TSS1500 / body	chr10:49863620-49864601 N-shore
cg24145616	-0.1501	$\mathbf{1.58*10^{-8}}$	-0.0788	$1.79*10^{-6}$	-0.0667	$3.51*10^{-4}$	-0.0403	0.0161	*GATA6* body	Unknown
cg25079060	-0.1025	$\mathbf{3.34*10^{-8}}$	-0.0517	$7.35*10^{-6}$	-0.0524	$5.59*10^{-5}$	-0.0215	0.0666	*RERE* 5' UTR / body	-
cg10751207	0.1233	$\mathbf{4.24*10^{-8}}$	0.0577	$3.83*10^{-5}$	0.0579	$2.45*10^{-4}$	0.0373	$8.37*10^{-3}$	*ATP9B* body	Unknown
cg07937962	-0.0399	$5.33*10^{-6}$	$-3.01*10^{-3}$	0.584	-0.0339	$\mathbf{1.95*10^{-8}}$	-0.0143	$8.86*10^{-3}$	*CXCL5* TSS1500	chr4:74864113-74864329 S-shore
cg06517429	0.0566	0.0209	0.0180	0.234	0.0384	0.0238	0.0107	0.482	*CASP7* 5' UTR / 1st exon	chr10:115439007-115440196 island
cg04129282	0.0595	$2.29*10^{-5}$	$6.08*10^{-3}$	0.487	0.0508	$1.56*10^{-7}$	0.0196	0.0249	*WDR93* TSS1500 *PEX11A* body	chr15:90233530-90234083 island
cg06772824	0.0480	$1.98*10^{-4}$	$6.44*10^{-3}$	0.420	0.0369	$3.64*10^{-5}$	0.0178	0.0258	*FAM176A* TSS200 / 5' UTR	chr2:75787717-75788312 island
cg03233931	0.0283	$1.87*10^{-4}$	$5.57*10^{-3}$	0.236	0.0208	$7.79*10^{-5}$	$8.95*10^{-3}$	0.0577	*LRRC20* TSS200 / 5' UTR / body	chr10:72141560-72142637 island
cg16274061	0.0751	$6.26*10^{-6}$	0.0174	0.0925	0.0557	$1.35*10^{-6}$	0.0216	0.0366	*SAP30L* TSS1500	chr5:153825417-153826526 N-shore
cg23560755	0.0475	$2.73*10^{-5}$	0.0126	0.0734	0.0315	$6.18*10^{-5}$	0.0146	0.0379	*SORBS3* body	chr8:22422534-22423702 N-shelf
cg00894435	0.0893	$1.29*10^{-5}$	0.0205	0.108	0.0652	$4.26*10^{-6}$	0.0272	0.0327	*SV2B* TSS1500	chr15:91642908-91643702

										island
Female infants										
cg14547404	0.2726	**$5.59*10^{-11}$**	0.1172	$4.17*10^{-5}$	0.1357	$2.92*10^{-5}$	0.1280	$3.89*10^{-6}$	*ARHGAP22* TSS1500 / body	chr10:49863620-49864601 N-shore
cg00670246	0.3707	**$8.38*10^{-11}$**	0.1565	$6.74*10^{-5}$	0.2047	$3.72*10^{-6}$	0.1508	$8.32*10^{-5}$	*GRHL1* body	-
cg09718046	-0.0844	$5.89*10^{-5}$	$4.22*10^{-3}$	0.767	-0.0860	**$2.76*10^{-8}$**	-0.0423	$2.03*10^{-3}$	-	-
cg07686628	-0.0930	$8.16*10^{-6}$	-0.0310	0.0280	-0.0846	**$4.54*10^{-8}$**	-0.0180	0.193	-	chr4:9533766-9534520 S-shelf
cg23628855	-0.0732	$1.57*10^{-3}$	-0.0213	0.171	-0.0923	**$5.17*10^{-8}$**	$6.38*10^{-3}$	0.674	*MYO3B* body	-
cg14561322	0.1308	$3.06*10^{-6}$	0.0552	$3.55*10^{-3}$	0.0909	$1.81*10^{-5}$	0.0400	0.0312	-	-
cg02737288	0.1481	$2.83*10^{-6}$	0.0457	0.0335	0.0989	$3.69*10^{-5}$	0.0664	$1.41*10^{-3}$	*SGPP1* body	Unknown
cg14262371	0.0281	$5.42*10^{-4}$	$2.78*10^{-3}$	0.612	0.0278	$4.08*10^{-6}$	0.0105	0.0483	*MOV10* 5' UTR / 1st exon	chr1:113217475-113218097 N-shore
cg20654468	0.0710	0.0187	-0.0379	0.0585	0.0472	0.0379	0.0910	$1.69*10^{-6}$	*LPXN* body	chr11:58345673-58347321 N-shelf

CpG sites that were significant also in multi-pollutant models are marked with *italics*. P-values that reached the significance cutoff are highlighted with bold types. TSS1500, ~1.5kb upstream from the transcription start site (TSS). TSS200, ~200kb upstream from the TSS. UTR, untranslated region. N-shelf, ~4kb upstream from the CpG island. N-shore, ~2kb upstream from the CpG island. S-shore, ~2kb downstream from the CpG island. S-shelf, ~4kb downstream from the CpG island.
